# Immunomodulatory Function of Vitamin D and Its Role in Autoimmune Thyroid Disease

**DOI:** 10.3389/fimmu.2021.574967

**Published:** 2021-02-19

**Authors:** Rui Zhao, Wei Zhang, Chenghong Ma, Yaping Zhao, Rong Xiong, Hanmin Wang, Weiwen Chen, Song Guo Zheng

**Affiliations:** ^1^Department of Endocrinology, Qujing Affiliated Hospital of Kunming Medical University, Kunming, China; ^2^Division of Immunology and Rheumatology, Ohio State University Wexner Medical Center and College of Medicine, Columbus, OH, United States

**Keywords:** vitamin D, immunomodulatory function, autoimmune thyroid disease, Hashimoto autoimmune thyroiditis, Grave's disease

## Abstract

Vitamin D is one of the most important nutrients required by the human body. It is a steroid hormone that plays an important role in regulating calcium and phosphorus metabolism, and bone health. Epidemiological studies have revealed a close correlation between vitamin D and many common chronic diseases. Additionally, vitamin D has recently been shown to act as an immunomodulatory hormone, and, accordingly, vitamin D deficiency was uncovered as a risk factor for autoimmune thyroid diseases, although the underlying mechanisms are still unknown. It is therefore necessary to disclose the role and mechanism of action of vitamin D in the occurrence and development of autoimmune thyroid diseases. This knowledge will help design intervention and early treatment strategies for patients with autoimmune thyroid diseases who present with low levels of vitamin D.

## Introduction

Vitamin D plays an important role in maintaining calcium and phosphorus balance, as well as bone health (1). Osteoporosis is a common manifestation of diseases affecting bone health due to an imbalance between osteoclast and osteoblast functions (2, 3). Vitamin D also plays an important part in many non-skeletal diseases (4, 5). Due to its roles in diabetes, cancer, autoimmunity, inflammatory responses, as well as muscle and cardiovascular disorders, it has gained increasing interest (6, 7). Such as Bouchemal et al. have mentioned the important role vitamin D plays in metabolic syndrome (8). Djeraba et al. have shown vitamin D deficiency was associated with active Behcet's disease (BD) and after the treatment of vitamin D down-modulates nitric oxide production in BD (9). We also previously reported that vitamin D deficiency is also a risk factor for neuropsychiatric disorders and autoimmune diseases (6). In addition, vitamin D, as it also appears to regulate the immune system, is a newly identified immunomodulatory hormone (10, 11). Among some common thyroid diseases, such as Hashimoto's thyroiditis (HT) and Graves' disease (GD), there are often autoimmune factors involved in their development (12). Current studies have found that the vitamin D receptor (VDR) is expressed on immune cells, where it can regulate their proliferation and differentiation, resulting in thyroid damage, and vitamin D has been shown to be associated with thyroid disease (13, 14). The underlying mechanisms, however, are not yet clear. We here aim to summarize the immunomodulatory effects of vitamin D in autoimmune thyroid disease (AITD) to understand how vitamin D exerts its immunomodulatory role in the body, and its influences and mechanisms in AITD.

## Immunomodulatory Mechanisms of Vitamin D

### Vitamin D Receptor

Vitamin D receptor (VDR) is an important signaling molecule for mediating downstream biological effects of active vitamin D. In the context of vitamin D-binding protein-dependent transport, 1,25(OH)2D3 binds to VDR on cells of the target organ to form a heterodimeric complex. There, the 1,25(OH)2D3-VDR-RXR complex exerts its biological function through the following mechanism: it specifically binds to the promoter regions of target genes on the DNA, thus regulating transcriptional activity of these target genes (15). Previous research has shown that dendritic cells, macrophages, T cells, B cells, and other immune cells express VDR (16). Hence, vitamin D can affect immune cells by binding to VDR, and immune cells can also metabolize 1,25(OH)2D3, which is the active form of vitamin D. In accordance with this, abnormal immune cells and inflammatory cytokines impact bone health (17, 18). Therefore, vitamin D has a regulatory effect on the body's innate and adaptive immune mechanisms.

### Effect of Vitamin D on Monocytes/Macrophages

The change of vitamin D nutritional status has a great influence on innate immunity mediated by monocytes in response to infection. Vitamin D may be involved in inducing autophagy, and its deficiency may be responsible for impaired antimicrobial activity of monocytes. Studies have found that macrophages isolated from mice that had been fed a diet deficient in vitamin D featured impaired phagocytosis, reduced chemotaxis, decreased abilities to release anti-inflammatory cytokines and respond to inflammatory stimuli, and reduced tumor-killing activity. In addition, mice deficient in vitamin D showed low expression of macrophage-specific antigen, but it could be rescued by supplementation with active vitamin D (13). This study confirmed that vitamin D is a key factor in macrophage maturation. Moreover, vitamin D could promote differentiation of monocytes to macrophages, enhance both phagocytosis and chemotaxis, and increase anti-tumor activities of mononuclear macrophages. Toll-like receptors (TLRs) can identify pathogen-associated molecular patterns, and consequently induce immune responses. Vitamin D can inhibit the expression of TLR2 and TLR4 on the surface of monocytes, resulting in a state of insufficient immune responsiveness and reduced production of inflammatory cytokines, such as TNF-α, and prevent overactivation of TLRs to inhibit inflammation (19). Indeed, TNF-α plays an important role during inflammation, and anti-TNF-α antibody has a therapeutic benefit in humans suffering from the diseases stated above (20–24).

### Effects of Vitamin D on Antigen-Presenting Cells (APCs)

APCs constitute a class of cells that can process antigens and present them to T cells, while being bound to major histocompatibility complex (MHC) molecules (25). Dendritic cells (DCs) represent the most powerful APC type. DCs can induce both innate and adaptive immune responses, and balance immune tolerance with immune activation (26, 27). Some studies have described the effects of 1,25(OH)2D3 on the morphology and function of DCs. Vitamin D can regulate the secretion of interleukins (ILs) by inhibiting the activation of p38 mitogen-activated protein kinase (MAPK) and nuclear factor-kB (NF-kB) signaling pathways in DCs. Then, vitamin D increases the production of anti-inflammatory cytokines, such as IL-10, and T-cell inhibitory molecules (programmed death-1, PD-1), and inhibits the secretion of pro-inflammatory cytokines IL-12, IL-23, TNF-α, and IFN-γ, which are involved in the differentiation of helper T (Th)1 and Th17 cells (28–31). In addition, DC-derived stimulatory signals are essential factors for activation of naïve T cells. Vitamin D can inhibit the maturation and differentiation of DCs, thus down-regulating the expression of the co-stimulatory molecules CD40, CD80, and CD86, as well as MHC class II molecules. Due to the lack of the mature DC surface molecules necessary, antigen-specific T cells are less responsive, or not at all (32). In line with this, 1,25(OH)2D3 inhibits the maturation of DCs, leading to reduced antigen uptake and activation of naïve T cells, which is an important process for suppressing autoimmunity.

### Effects of Vitamin D on T and B Cells

It has been previously reported (33) that naive and memory T cells express VDR, which indicates that vitamin D can act directly on T cells and regulate T cell responses. Moreover, vitamin D can affect the distribution of Th1, Th2, and Th17 cells, consequently altering the functional subclasses of T cells. At present, studies have provided evidence that vitamin D can inhibit the conversion of antigen- and lectin-stimulated naïve CD4+ T cells to Th1 cells, and that vitamin D can also inhibit the production of IL-2 and IFN-γ by Th1 cells, decrease the secretion of IgG by B lymphocytes, and at the same time promote the transformation of Th1 to Th2 cells, and up-regulate the activity of the latter. Vitamin D promotes secretion of IL-4, IL-5, and IL-10 from Th2 cells, regulates Th1/Th2 cell ratios, and limits potential tissue damage caused by Th1-induced immune responses (34). Th17 cells constitute a newly identified subset of T cells (35). They have a pro-inflammatory effect in many inflammatory diseases (36–38), as they can secrete many cytokines, such as IL-17A, IL-17F, IL-21, IL-22, and IL-6, and bind CC-type chemokine 6 (CCL-6) and TNF-α. They participate in autoimmune disease processes and perform specific functions. IL-17 and IL-22 receptors are widely distributed, and play a synergistic and pro-inflammatory role, which may be the basis for inducing tissue inflammation and autoimmunity in Th17 activation. We and others provided evidence that vitamin D can inhibit both the differentiation of CD4+ T cells to Th17 cells and the secretion of IL-17 from Th17 cells (11, 39). Vitamin D also promotes the differentiation of naive CD4+ T cells to regulatory T cells (Treg). Unlike other effector T cells, Treg cells can regulate the body's immune response by regulating cytokine secretion and T cell activation (40–42).Vitamin D can also directly affect the proliferation of Treg cells without inhibiting their function, and promote the secretion of IL-10 by Treg cells (43, 44). Therefore, vitamin D plays an important role in autoimmune diseases and hosts transplant rejection. The reduction of Th17 cells usually coincides with the increase of Treg cells. An important focus of improving the treatment of autoimmune diseases is to restore the Th17/Treg imbalance that occurs during chronic inflammation. Vitamin D can regulate the Th17/Treg imbalance by up-regulating Treg cells and inhibiting Th17 cell differentiation (45).

B cells also express VDR, but the effect of vitamin D on B cells is still controversial. One the one hand, vitamin D can induce low reactivity of B cells, or it can cause B cell apoptosis directly, inhibit the production of memory B cells, and reduce the production of immunoglobulins. One the other hand, 1,25(OH)2D3 may alter CD4+ T-cell responses, or suppress cytokine secretion by inhibiting the actions of monocytes/macrophages, thereby indirectly inhibit the functions of B cells (11). Indeed, the B cell response depends upon T cell help (46, 47). B cell surfaces also express enzymes involved in vitamin D metabolism, including 1-α-hydroxylase and 24-hydroxylase, indicating that vitamin D can affect the functions of B cells (33). Therefore, vitamin D has a potential role in diseases with B cell dysfunction. To sum up, vitamin D plays an important role in the occurrence and development of autoimmune diseases. Vitamin D can enhance innate immunity and regulate adaptive immune function.

## Vitamin D Deficiency and HT

HT, also known as Hashimoto's disease or chronic lymphocytic thyroiditis, is an AITD with unknown etiology. Current studies have shown that Th1/Th2 cell imbalance and Th1 cell activity enhancement are the main pathogenic factors of HT (48). Vitamin D may play an immunosuppressive role by inhibiting multiple parts of the HT immune response (49, 50), such as: (1) vitamin D binding to its receptor to prevent DC-dependent T cell activation that then reduces the production of pro-inflammatory cytokines (IL-2, IL-5, IL-17, and TNF-α), and decreasing the cytokine-mediated immune response; (2) vitamin D inhibiting autoimmune thyroiditis through down-regulating HLA class II gene expression in the thyroid, thus inhibiting lymphocyte proliferation and secretion of inflammatory cytokines; (3) vitamin D deficiency causing a large number of B cells to proliferate and differentiate into plasma cells, causing the latter to secrete large amounts of IgG, IgE, and other immunoglobulins, which eventually cause damage to thyroid cells and then trigger HT; and (4) vitamin D inhibiting differentiation of naïve T cells into Th17 cells, and suppressing the secretion of Th17-derived cytokines IL-17 and IL-21, while promoting the metabolism of Treg cells to restore the balance of the body's Th17/Treg cell ratio, and inhibit Th17 cell-induced thyroid inflammation (51).

A case-control study (52) found that each increase in serum 25(OH)D levels by 5 nmol/L reduced the risk of HT by 1.62 times. A study by Bozkurt et al. (53) showed that serum 25(OH)D levels in HT patients were significantly lower than those in the control group, and the severity of 25(OH)D deficiency was associated with the duration of HT and thyroid antibody levels. Others have demonstrated that serum 25(OH)D is negatively correlated with serum anti-TPO antibody in HT patients, which was found to be significantly reduced after 4 months of vitamin D supplementation in HT patients [daily oral administration of 1,200–4,000 IU vitamin D3, with the goal of 25(OH)D concentrations of up to 40 ng/mL] (54). A large number of studies (55, 56) have also documented that vitamin D deficiency prevails in HT patients, that vitamin D is involved in the occurrence and development of HT, and that vitamin D supplementation helps to alleviate HT conditions. Botelho et al. examined 88 patients with HT and 71 euthyroid healthy subjects for vitamin D status and thyroid autoimmunity markers, as well as their relationship with cytokines produced by Th1, Th2, and Th17 cells. They found an association between vitamin D and TNF-α, IL-5, and IL-17 levels in these patients, which indicated the relevance of this relationship with autoimmunity in HT (57). Nodehi et al. assessed the frequencies of Th1, Th17, Th2, and Treg cells and the levels of IFN-γ, IL-17, IL-4, and IL-10 in 34 HT patients, among which 17 patients were treated weekly with 50,000 IU of cholecalciferol and the rest were treated with placebo through oral administration for 3 months. They concluded that vitamin D supplementation provides beneficial immunological effects in HT patients through decreasing Th17/Treg cell ratio (58). However, controversial results have been reported (59). Zhang et al. concluded that vitamin D status was not associated with positive thyroid autoantibodies, but higher 25(OH)D levels were associated with lower TSH levels (60). Moreover, D'Aurizio et al. revealed that there was no differences in vitamin D deficiency and 25(OH)D levels between 100 AITD patients and healthy controls (61). Similarly, Yasmeh et al. reported that there was no positive correlation between vitamin D and TPOAb levels in HT males relative to the control group, thereby concluding that HT is not associated with higher rates of vitamin D deficiency relative to the control group (62). Therefore, more research data are still needed to verify the relationship between vitamin D deficiency and HT, which will help to determine whether vitamin D supplementation is effective for HT treatment.

## Vitamin D Deficiency and GD

GD, known as diffuse toxic goiter, is the most common cause of hyperthyroidism, and its pathogenesis is complex and unclear. Two groups have demonstrated (63, 64) that vitamin D inhibits the pathogenesis of GD through the following immunomodulatory mechanisms: (1) vitamin D inhibits the differentiation and maturation of DCs, decreases the secretion of the pro-inflammatory cytokines IL-2, IL-23, and IL-12 by DCs, and blocks hyperthyroidism caused by autoimmune responses; (2) vitamin D can directly act on Th1 and Th2 cells, and inhibit the occurrence of autoimmune responses by inhibiting Th1 cells, and by upregulating the activity of Th2 cells and the level of cytokines they secrete; and (3) vitamin D inhibits B cell proliferation, plasma cell differentiation, immunoglobulin secretion, and memory B cell production, and is associated with the onset of GD.

The aforementioned case-control study (52) also noted that each increase in serum 25(OH)D levels by 5 nmol/L reduced the risk of GD by 1.55 times. Zhang et al. (65) have shown that the lower the vitamin D level, the higher the serum thyrotrophin receptor antibody (TRAB) level in GD patients. These findings suggest that vitamin D deficiency may be one of the risk factors contributing to the onset of GD. Ahn et al. examined patients diagnosed with Graves' disease and treated with ATDs for more than 1 year after the discontinuation of ATD. This study have shown that low serum 25(OH)D levels are highly correlated with the incidence of GD recurrence, and that serum 25(OH)D may be an independent risk factor for predicting the recurrence of GD after the discontinuation of anti-thyroid drug (ATD) treatment (66). In this regard, opposing arguments have also been made. Ke et al. reported low serum vitamin D levels in HT patients, which did not alter significantly in GD patients compared with non-AITD controls (67). However, Cho et al. demonstrated that there was no beneficial effect of daily supplementation of vitamin D on the recurrence of Graves' disease within 1 year after the discontinuation of ATD. However, the time of recurrence was delayed in patients who reached sufficient levels of vitamin D after supplementation (68).

## Conclusions

In recent years, there have been more and more studies on the action of vitamin D in non-skeletal diseases, especially on the regulatory effect of vitamin D on immune function. As a hormone of immune regulation, vitamin D has become a research focus. Current epidemiological statistics and clinical studies support an association between vitamin D and AITD, where vitamin D deficiency increases the risk of AITD. At present, some literatures have reported that vitamin D treatment can improve the condition of patients with AITD, but whether it can prevent AITD has not been studied. In addition, because vitamin D levels in the body are affected by many factors, results reported by various studies are inconsistent. Whether vitamin D supplementation can help prevent and/or treat thyroid disease, deserves a clinical trial in multiple centers in the future. Recently, researchers have proposed that the skeletal health is maintained at vitamin D level of 30–40 ng/mL, but higher vitamin D levels does not exert better effects. Vitamin D levels higher than 40 ng/mL tend to loose their beneficial effects on bone. If supplementing vitamin D is considered to be useful for AITD patients, it is important to address the following questions: how to supplement vitamin D, and monitor vitamin D level and thyroid function, and at what level can vitamin D improve the condition of patients with AITD? Therefore, these questions are worth exploring.

## Author Contributions

WC: conceptualization and supervision. WZ: validation, visualization, and project administration. HW: formal analysis. CM, YZ, and RX: investigation. RZ: data curation and writing—original draft preparation. SZ: writing—review and editing. All authors contributed to the article and approved the submitted version.

## Conflict of Interest

The authors declare that the research was conducted in the absence of any commercial or financial relationships that could be construed as a potential conflict of interest.
